# Synthesis and activity of conjugates Gallic acid - GnRH-III

**DOI:** 10.1186/1753-6561-8-S4-P41

**Published:** 2014-10-01

**Authors:** Júlia Pinto Piccoli, Eduardo Maffud Cilli

**Affiliations:** 1 UNESP, Universidade Estadual Paulista, Instituto de Química, Araraquara, SP, Brazil

## Background

Most of scientific studies with peptide have aim to therapeutic use. New compounds have the ability to activate or inhibit biological processes. The combination of anticancer drugs with a molecule which recognizes tumor specific connections, through a receiver, may provide an effective anticancer agent. The hormone GnRH-III, analogue of the hormone GnRH (Gonadotropin releasing hormone), has been described as inhibitor of tumor growth, and has the possibility of use in cancer therapy. In addition, the gallic acid and its derivatives show great potential biological, inducing apoptosis in tumor cells [1]. The aim of this work was to study the feasibility of synthesis of conjugated containing Gallic Acid and the hormone GnRH-III in order to obtain anticancer molecules and available the activity

## Methods

GnRH-III (Glp-His-Trp-Ser-His-Asp-Lys-Pro-Trp-Gly-NH2) was synthesized using a technique called solid-phase peptide synthesis (SPPS) [2]. The coupling of Fmoc-amino acid and gallic acid were performed by activation of carboxyl groups with diisopropylcarbodiimide (DIC) / N-hydroxybenzotriazole (HOBt). We used the protector Trt (trityl) to protect the ε-amino group of Lys, which allowed selective deprotection of this group for subsequent coupling in the side chain of Lys. Purification of the crude material was carried out in a system of high performance liquid chromatography (HPLC). The purity of the fractions was determined on an analytical HPLC and confirmed by mass spectrometry. Mosman colorimetric test was performed in order to detect biological activities

## Results and conclusions

The synthesis of the conjugate containing gallic acid proved to be difficult, but using optimized conditions of deprotection of the ε-amino group of Lys, the conjugated GnRH-III/gallic acid was obtained. According to the results, it can be noted that the entrance of gallic acid in question was not a major problem encountered in synthesis, but the removal of the protecting group of Lys. The results showed, as expected, that the IC_50 _of gallic acid was 14 ug/mL. The conjugate GnRH-III/Gallic Acid showed IC_50 _much smaller than gallic acid. Apoptosis was induced at low concentrations, showing an interesting compound for the anticancer research.
